# Fracture Dislocation of the Pisiform Bone in 14-Year-Old Boy—A Case Report

**DOI:** 10.3390/medicina60040532

**Published:** 2024-03-25

**Authors:** Ondřej Procházka, Tomás Sánchez, Karolína Kašpárková

**Affiliations:** Department of Hand Surgery, Canton Hospital Olten, 4600 Olten, Switzerland; tomas.sanchez@spital.so.ch (T.S.); karolina.kasparkova@insel.ch (K.K.)

**Keywords:** pisiform bone, pediatric, fracture, dislocation, luxation, excision

## Abstract

We present the case of a 14-year-old patient who suffered fracture dislocation of the pisiform bone (PB) along with fractures of the scaphoid, proximal radius, and proximal phalanx of the thumb due to high-energy trauma directly to the extended wrist. This combination of fractures has not been previously reported in the literature. Currently, there is no consensus in the literature regarding the optimal treatment approach for such cases. In our management, initial attempts at closed and open reduction were unsuccessful, leading to the decision for primary pisiformectomy. Our report includes a follow-up of 3.5 years, demonstrating a very good outcome. Based on this case and a few similar published cases, primary pisiformectomy appears to be a viable and well-accepted option, particularly among young patients. Additionally, we conducted a review of radiographic criteria and management strategies for this specific injury and related conditions.

## 1. Introduction

The pisiform bone (PB), the smallest carpal bone, typically ossifies between ages 7 and 10 and completes ossification by age 12 [1]. It is considered a sesamoid bone within the flexor carpi ulnaris (FCU) tendon and part of the proximal carpal row [2]. While it articulates flatly with the triquetrum without contributing to stability, its stability relies on soft tissue attachments such as the pisohamate and pisometacarpal ligaments, joint capsule, extensor retinaculum, FCU tendon, abductor digiti minimi muscle, and the transverse carpal ligament (TCL) (Figure S1).

While the precise function of the pisiform remains uncertain, it serves as a central point for soft tissue attachment on the medial wrist and functions as a lever, akin to the patella, enhancing wrist flexion force. The soft tissue convergence over the pisiform enables sub-periosteal excision and repair without disrupting the insertion of the flexor carpi ulnaris (FCU) [2].

Simultaneous fracture and dislocation of the pisiform bone is exceedingly rare in both children and adults, with only a few reported cases in pediatric literature and no established consensus on treatment. Upon comparison with existing literature through a PubMed database search, we identified four pediatric patients with this condition [3,4,5]. Additionally, our analysis encompassed nine patients with simple dislocations [6,7,8,9,10,11,12,13,14] and one patient [15] with isolated fractures of the pisiform bone from available reports of similar injuries.

## 2. Case Report

A 14-year-old male patient was admitted to the emergency department following a snowboarding accident, where he fell at high speed, resulting in hyperextension of his right wrist, estimated at about 60 km/h. Upon examination, there was noticeable swelling in the wrist without any apparent skin injury. Painful palpation was noted over the distal radius, pisiform bone, and proximal thumb, although no neurovascular deficits were detected. Limited wrist motion was observed due to pain. Additionally, the patient experienced localized painful palpation over the radius head, although his right elbow exhibited normal range of motion upon comparison with the unaffected site. Notably, the patient denied any prior problems to the affected hand, and otherwise, he was in good health with no current medications.

**Figure 1:**
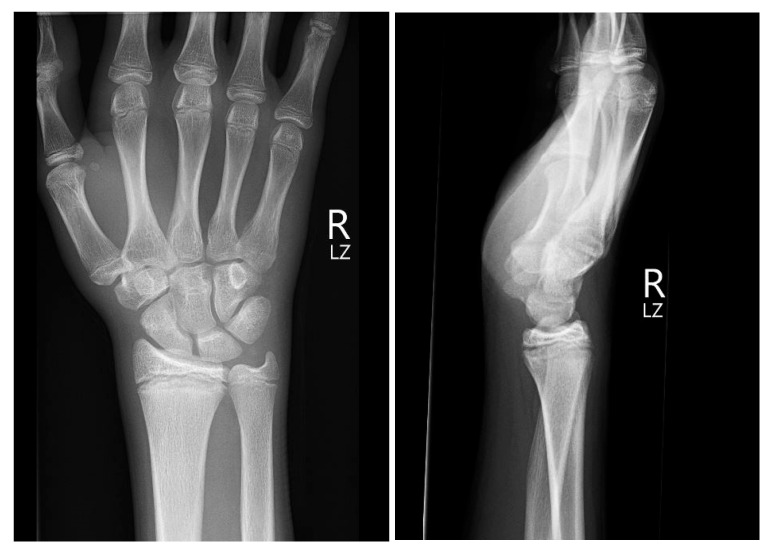
Initial X-ray views in ap (**left**) and lateral (**right**) planes.

**Figure 2:**
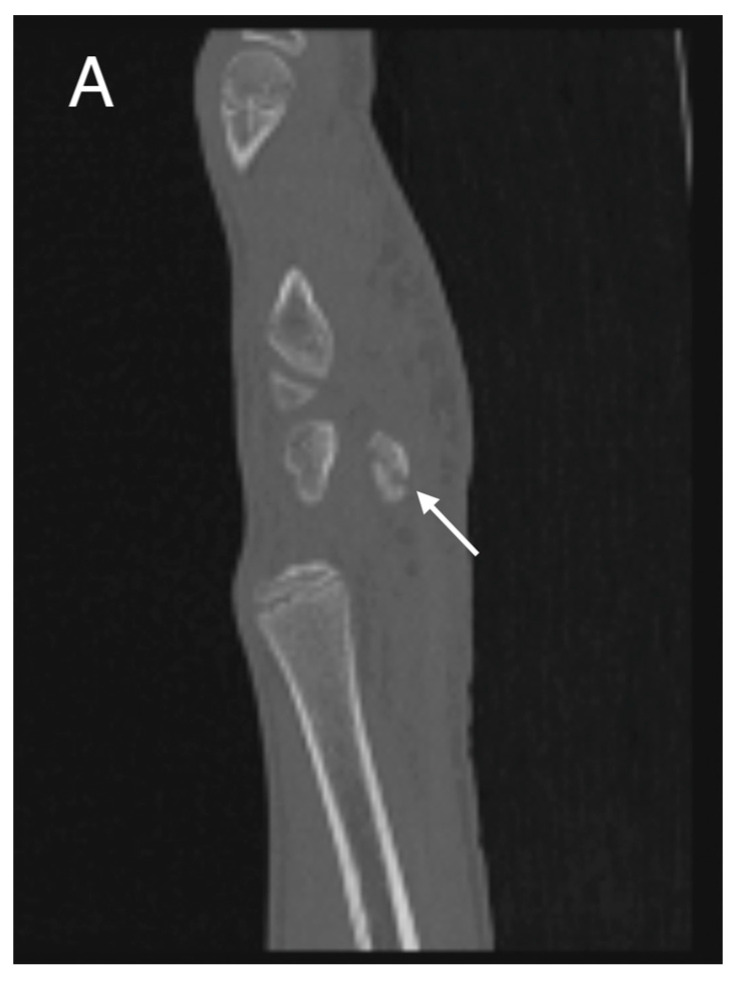

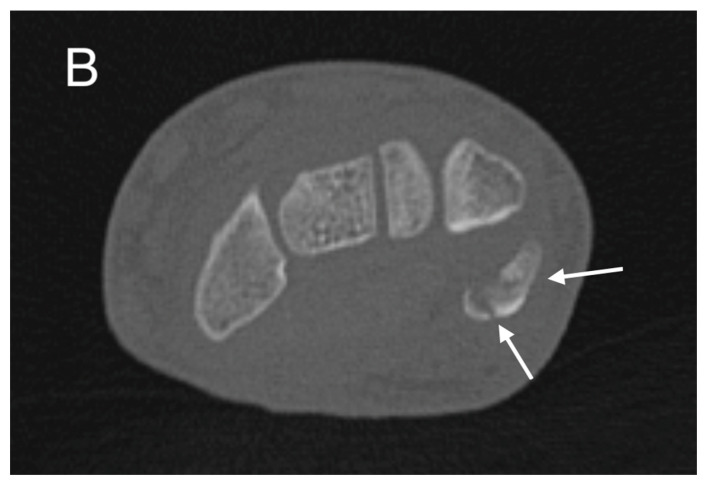
Initial CT study with sagittal (**A**) and axial (**B**) images showing a non-displaced fracture of the pisiform bone (white arrows).

Initial conventional radiographic assessment showed no definitive fracture, with a potential suspicion of a proximal radius fracture (Figure 1). Due to significant tenderness over the radial fossa and pisiform, a CT scan was performed, confirming a nondisplaced scaphoid fracture and a suspected pisiform fracture with dislocation (Figure 2).

**Figure 3:**
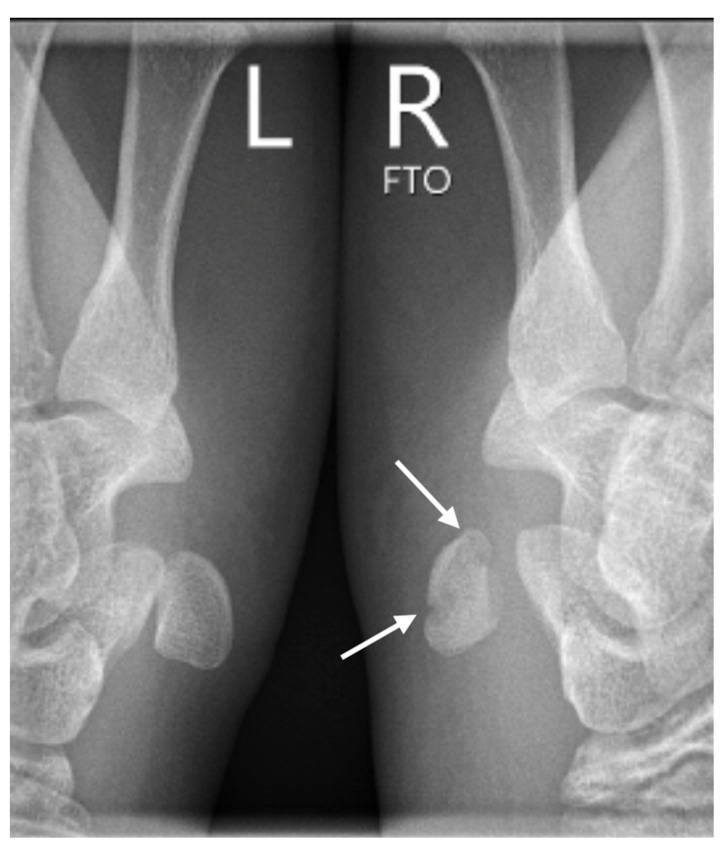
The semisupination oblique X-ray in 30° angle, side-by-side comparison (L: left, R: right), showing a non-displaced fracture of the pisiform bone (white arrows).

Further confirmation of the dislocation was obtained through subsequent conventional radiographs, specifically utilizing the semi-supination oblique view in a 30° angle (pisiform, pisotriquetral view), with side-by-side comparison of both wrists (Figure 3).

The procedure was conducted under general anesthesia. 

Acknowledging our preference for less invasive interventions, our initial strategy encompassed arthrocentesis via an ulnar approach, followed by hemarthrosis aspiration. Although we proposed a simplified repositioning technique, it lacked validation in the existing literature. Subsequently, despite earnest attempts, both closed reduction and the “Joystick maneuver” utilizing a 1.25 mm K-Wire insertion into the pisiform bone were futile. Consequently, we pivoted towards a more direct approach, opting for an open ulnar palmar procedure [16]. This entailed making a longitudinal incision centered over Guyon’s canal to effectively address the intricate nature of the fracture dislocation. Upon releasing the neurovascular bundle and dissecting until ligaments and bone were exposed, we identified multiple fractures of the pisiform bone. 

**Figure 4:**
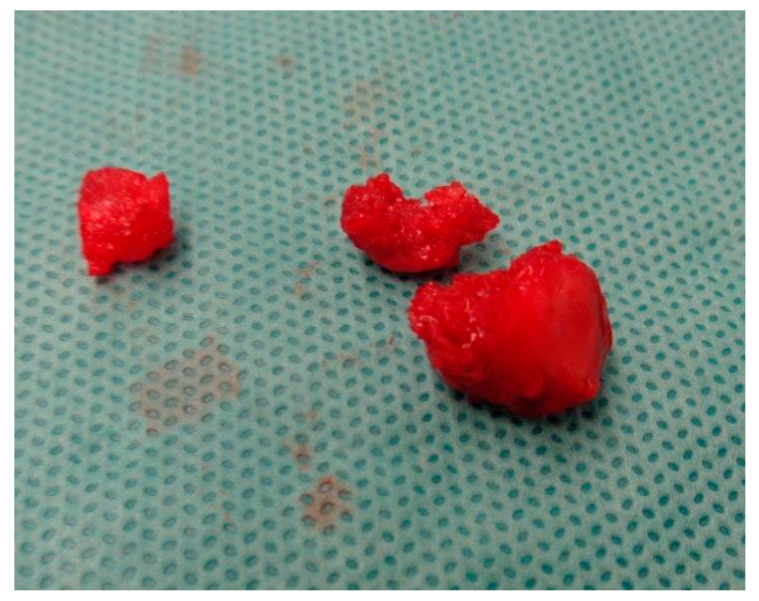
Extracted pisiform bone, in three parts.

The pisotriquetral joint (PTJ) capsule was interposed in such a manner that it hindered the reduction process, making it unachievable. As observed in the CT scan, the fracture was in three parts, further complicating the situation. Despite our efforts to address this challenge by partially removing the joint capsule, the complexity and instability of the fracture persisted, impeding our attempts at achieving a stable reduction. While our initial plan was to proceed with screw stabilization in the event of a stable reduction, it is worth noting that there has been only one documented case in the literature involving K-wire stabilization up to this point [5]. Consequently, osteosynthesis was deemed unfeasible under these circumstances. Therefore, we ultimately decided to proceed with a pisiformectomy (Figure 4).

**Figure 5:**
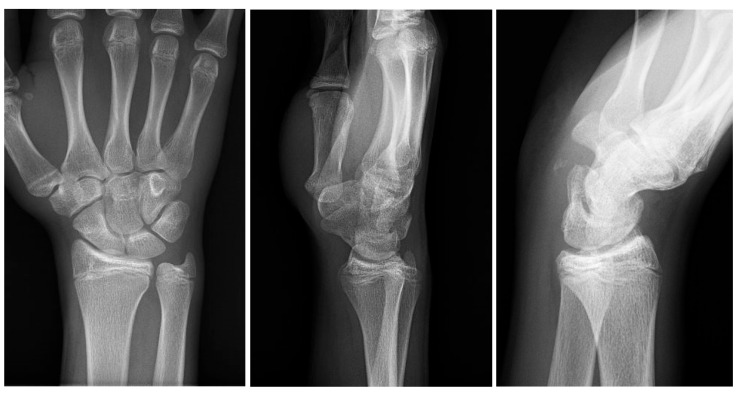
The follow-up X-ray (anteroposterior view on the **left**, lateral view in the **middle**, and 30° supination view on the **right**) after surgery revealed only small remaining fragments of the pisiform bone (depicted on the **right** image).

For the treatment of both the scaphoid fracture and distal radius fracture, we opted for a conservative approach involving immobilization of the wrist and thumb without immobilization of the IP joint of the thumb in a plaster, for a duration of 6 weeks. Upon follow-up X-ray after surgery, only small residual fragments of the pisiform bone were observed (Figure 5), with no evidence of secondary dislocation of the scaphoid or phalangeal fracture of the thumb. Physical therapy, involving gentle mobilization of the wrist, commenced immediately following the removal of the plaster cast. Given the absence of tenderness over the fractured bones, we decided to initiate exercises under loading in the subsequent 2 weeks.

The next follow-up appointment took place 12 weeks after the surgery. At this point, after a full 12 weeks of recovery, our patient reported being completely symptom-free. Although there was a slight deficiency noted in the range of motion and strength of the wrist, it was minimal. Importantly, our patient experienced no discomfort or inconvenience associated with the absence of the pisiform bone.

During the follow-up examination 3.5 years after the injury, the patient exhibited no symptoms, achieving a remarkable DASH score of 3.3/100 and a Mayo Wrist score of 95. These scores indicate excellent functional outcomes and minimal impact on daily activities and wrist function, underscoring the success of the treatment approach and the patient’s remarkable recovery.

## 3. Discussion

The initial report of a pisiform fracture dates back to 1847 by Guibout [17]. Pisiform bone injuries present a notable challenge, especially among pediatric patients, given the scarcity of the literature available. To address this gap, we broadened our review to include similar conditions, aiming to enhance our understanding of management strategies and contribute to the overall knowledge in this area.

The primary challenge lies in diagnosis, as changes on X-rays are often subtle or absent in pisiform bone injuries. Fractures of PJ may go unnoticed, especially in patients younger than 7.5 years old, largely due to the absence of the ossification center [18]. Hence, a CT scan may be necessary for accurate assessment. Typically, these injuries coincide with more severe fractures or soft tissue damage, with diagnosis guided by the predominant issue. In our case, the pisiform fracture or dislocation was incidentally revealed during diagnostic evaluation for the primary concern. Due to suspicion of a concurrent dislocation of the PTJ, we conducted additional X-rays of both PTJ’s for a side-by-side comparison as a reference (Figure 4).

While specific radiological criteria for diagnosing pisiform dislocation have been proposed in adults, none have yet been defined for children. In adults, diagnosis typically requires conditions such as a joint space dilatation of 4 mm or more, or a PTJ surface dislocation of 2 mm or more [19,20]. We interpreted these criteria for our patient considering their proximity to skeletal maturity.

Unlike CT scans, MRI has the capability to identify both bone and cartilage fragments, particularly in pediatric cases [21]. Although MRI would have been beneficial for assessing soft tissue injury extension and identifying any interposed structures, we did not conduct one. The surgical outcomes validated the suspected nonunion.

According to the current literature, different treatment strategies are described, but there is no consensus of some “gold standard”. There is generally a tendency to reach a closed reduction.

If there are any signs of neurological injury to the ulnar nerve [13], emergency reduction of the pisiform bone is necessary. 

The reduction by direct pressure on the dislocated bone in the initial step appears to be an effective procedure in certain cases [3]. Regarding positioning, maximal flexion and pronation have been described as recommended maneuvers for repositioning [8]. Although there is no published literature describing this, we attempted extraction of the hemarthrosis as well. In cases where closed reduction attempts are unsuccessful, some authors suggest the joystick maneuver as the next option, which can be challenging, especially in the presence of a fractured bone [16]. If successful repositioning is achieved, open reduction and internal fixation (ORIF) with K-wires can be considered, as demonstrated by Giannetti’s one case with very good results [5]. The last resort appears to be pisiformectomy, a technique successfully employed in all young patients identified in the literature [7,9,15]. Pisiformectomy can also be performed later in symptomatic patients with posttraumatic PTJ osteoarthritis, yielding good results [22].

Carroll and Coyle conducted a study involving adults experiencing chronic pain in the pisiform area caused by malunion or nonunion (in three patients), subluxation, dislocation, or pisotriquetral arthritis [19]. After the resection of the pisiform bone, complete relief was achieved in 65 out of 67 patients, with minimal long-term disabilities reported.

In this case, despite unsuccessful closed reduction attempts, open ulnar palmar approach and pisiformectomy led to successful management, with conservative treatment for associated fractures resulting in sub-complete recovery and almost normal wrist function already at the 12-week follow-up. The result after 3.5 years seems to be, with a DASH score of 3.3/100 and a Mayo Wrist score of 95, excellent.

The literature contains only four reported cases of fracture dislocation of the pisiform bone in children (Table S1). Hurni documented a case involving an 11-year-old boy who sustained a distal radius fracture (Salter–Harris type 2) following a car accident, along with an additional fracture dislocation of the pisiform bone [3]. Closed reduction of both fractures was performed, followed by splinting in a slight extension using a long arm cast for 30 days. Post-treatment evaluation revealed stable pisiform bone with no tenderness, and the patient achieved an excellent overall outcome.

Mancini 2005 presented two cases, including a 13-year-old boy who experienced a radius fracture (Salter–Harris type 2) after a fall while running, coupled with a fracture dislocation of the pisiform bone with 7 mm of anterior dislocation [4]. Similar to Hurni’s approach, closed reduction (technique not specified) was performed, followed by casting for 30 days. Upon reevaluation 30 years later, the patient remained fully asymptomatic.

In another case described by Mancini 2005, a 12-year-old boy sustained a distal radius fracture (Salter–Harris type 1) with two fragments and a pisiform bone fracture dislocation, resulting in a 5 mm widening of the pisotriquetral joint [4]. Treatment consisted of closed reduction (technique not specified) followed by casting for 30 days. Upon reevaluation 43 years later, the patient remained asymptomatic, although no objective measurements were performed.

Additionally, Giannetti 2020 reported a case involving an 11-year-old boy who fell on a mountain trail, sustaining a greenstick fracture of the radius and a fracture of the ulna styloid [5]. A CT scan confirmed an additional fracture dislocation of the pisiform bone. Closed reduction of the pisiform bone was unsuccessful, leading to surgical intervention. The procedure involved percutaneous fixation of the radius fracture followed by a volar approach for manual repositioning of the pisiform bone and fixation with a 1.2 mm K-wire to the triquetrum and hamatum bones. The ruptured ligaments (pisotriquetral, pisohamate, and pisometacarpal) were sutured, resulting in anatomical repositioning. The K-wire was removed, and a postoperative splint was applied one month after surgery. Two months post-surgery, the patient achieved full range of motion without pain or discomfort, with a Mayo Wrist score of 95.

We have identified an additional nine patients with simple dislocation of the pisiform (Table S1), four of whom underwent treatment with closed reduction and splinting [6,8,10,14]. Remarkably, they all reported being asymptomatic within 2 to 3 weeks following treatment. Another two patients [11,12] underwent open reduction surgery accompanied by splinting for a duration of 6 weeks. Notably, Choong 2023 presented the case of a 15-year-old patient who experienced transient ulnar palsy, which resolved completely within two weeks postoperatively [12]. Normal function was reported in all three cases after a period of 2 months. Furthermore, Korovessis (1983), McCarron RF (1989) and Pevny (1996) discussed the cases of three patients who underwent pisiformectomy after initial unsuccessful attempts at closed reduction [7,9,13]. Encouragingly, all three patients were asymptomatic after a 2-month follow-up.

In 2015, Brouwers documented the case of a 9-year-old boy with non-union of the pisiform bone, after missed diagnosis [15]. He was treated 10 months after the injury with excising of the superficial, non-united pole of the pisiform bone, resulting in an excellent outcome 1 year after the surgery.

## 4. Conclusions

Our case study highlights the challenges of diagnosing and treating rare pisiform bone fractures in young patients with wrist injuries. Using advanced imaging like CT scans is crucial for accurate diagnosis. Removing the pisiform bone directly led to a successful outcome in our case, aligning with literature supporting its efficacy. This case underscores the importance of considering pisiform bone injuries in pediatric wrist trauma cases for thorough evaluation and appropriate management.

## Data Availability

Data is contained within the article or Supplementary Material.

## Supplementary Materials

The following supporting information can be downloaded at: https://www.mdpi.com/article/10.3390/medicina60040532/s1, Figure S1: Anterior view on stabilizing structures of the pisiform bone (right wrist) and schematic representation of their stabilizing function; Table S1: Documented cases of Pisiform bone injuries, encompassing dislocations, fracture dislocations, and fractures in patients up to 18 years of age.
