# Supplementary material for: Fracture Dislocation of the Pisiform Bone in 14-Year-Old Boy—A Case Report
Source: Medicina (Kaunas). 2024 Mar 25;60(4):532. doi: 10.3390/medicina60040532 (PMC11052046; doi:10.3390/medicina60040532)
Supplement: Supplementary file 1 [file medicina-60-00532-s001.zip › medicina-2916379-supplementary.pdf]

Suplementary Files

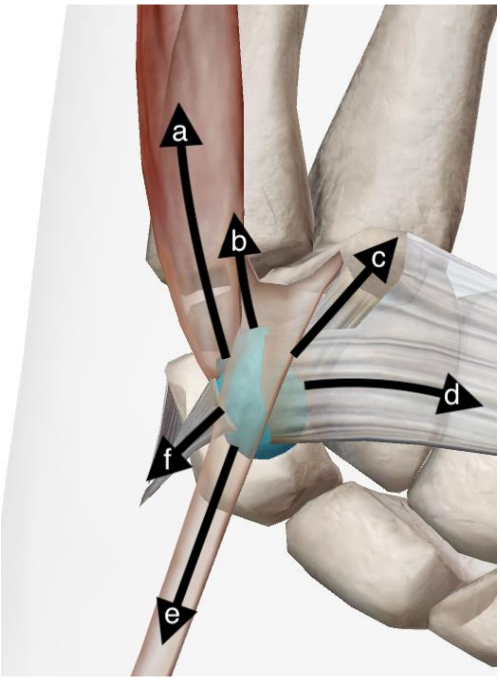

Figure S1. (Image courtesy of Visible Body), Right wrist: anterior view on stabilizing structures of the pisiform bone (blue) and schematic representation of their stabilizing function. a) abductor digiti minimi (ADM), b) piso-hamate ligament, c) piso-metacarpal ligament, d) transverse carpal ligament (TCL), e) flexor carpi ulnaris tendon (FCU), f) extensor retinaculum

Table S1: This table is showcasing documented cases of Pisiform bone injuries, encompassing dislocations, fracture dislocations, and fractures in patients up to 18 years of age.

Author	Year	Age	Sex	Kind of injury	Therapy	Result
Ashkan [6]	1998	9	W	Simple dislocation	Closed reduction	Asymptomatic
Browers [15]	2015	9	M	Nonunion	Partial excision	Asymptomatic
Cohen [14]	1922	11	M	Simple dislocation	Closed reduction	Mild symptoms after 2 Weeks, than lost in follow up
Giannetti [5]	2020	11	M	Fracture Dislocation	ORIF with K-Wire	Asymptomatic in 15 Months, Mayo Wrist score 95
Hurni [3]	2015	11	M	Fracture dislocation	Closed reduction	Asymptomatic
Choong [12]	2023	15	M	Simple dislocation	open reduction	Asymptomatic, Transient ulnar palsy resolved
Korovessis [9]	1983	14	M	Simple dislocation	Pisiformectomy after unsuccessful reduction	Asymptomatic
Kubiak [10]	2001	13	W	Simple dislocation	Closed reduction	asymptomatic
Letsch [11]	2016	16	M	Simple dislocation	open reduction	asymptomatic
Mancini [4]	2005	13	M	Fracture dislocation	Closed reduction	Asymptomatic
Mancini [4]	2005	12	M	Fracture dislocation	Closed reduction	Asymptomatic
McCarron [7]	1989	18	M	Simple dislocation	Pisiformectomy after unsuccessful reduction	Asymptomatic
Prochazka	2024	14	M	Fracture dislocation	Pisiformectomy after unsuccessful reduction	Asymptomatic with DASH score: 2.5/100 and Mayo Wrist Score 95
Pevny [22]	1996	17	M	Simple dislocation	Pisiformectomy after unsuccessful reduction	Asymptomatic
Sharara [8]	1993	18	M	Simple dislocation	Closed reduction	Asymptomatic
